# Primary DNA damage and genetic polymorphisms for *CYP1A1*, *EPHX *and *GSTM1 *in workers at a graphite electrode manufacturing plant

**DOI:** 10.1186/1471-2458-7-270

**Published:** 2007-10-01

**Authors:** Massimo Moretti, Marco Dell'Omo, Milena Villarini, Roberta Pastorelli, Giacomo Muzi, Luisa Airoldi, Rossana Pasquini

**Affiliations:** 1 Department of Medical-Surgical Specialities and Public Health, University of Perugia, Via del Giochetto, 06122 Perugia, Italy; 2 Institute of Occupational Medicine and Toxicology, University of Perugia, Via E. dal Pozzo, 06126 Perugia, Italy; 3 Department of Environmental Health Sciences, Istituto di Ricerche Farmacologiche "Mario Negri", Via Eritrea 62, 20157 Milan, Italy

## Abstract

**Background:**

The results of a cross-sectional study aimed to evaluate whether genetic polymorphisms (biomarkers of susceptibility) for *CYP1A1*, *EPHX *and *GSTM1 *genes that affect polycyclic aromatic hydrocarbons (PAH) activation and detoxification might influence the extent of primary DNA damage (biomarker of biologically effective dose) in PAH exposed workers are presented. PAH-exposure of the study populations was assessed by determining the concentration of 1-hydroxypyrene (1OHP) in urine samples (biomarker of exposure dose).

**Methods:**

The exposed group consisted of workers (n = 109) at a graphite electrode manufacturing plant, occupationally exposed to PAH. Urinary 1OHP was measured by HPLC. Primary DNA damage was evaluated by the alkaline comet assay in peripheral blood leukocytes. Genetic polymorphisms for *CYP1A1*, *EPHX* and *GSTM1* were determined by PCR or PCR/RFLP analysis.

**Results:**

1OHP and primary DNA damage were significantly higher in electrode workers compared to reference subjects. Moreover, categorization of subjects as normal or outlier highlighted an increased genotoxic risk OR = 2.59 (CI95% 1.32–5.05) associated to exposure to PAH. Polymorphisms in *EPHX* exons 3 and 4 was associated to higher urinary concentrations of 1OHP, whereas none of the genotypes analyzed (*CYP1A1*, *EPHX*, and *GSTM1*) had any significant influence on primary DNA damage as evaluated by the comet assay.

**Conclusion:**

The outcomes of the present study show that molecular epidemiology approaches (i.e. cross-sectional studies of genotoxicity biomarkers) can play a role in identifying common genetic risk factors, also attempting to associate the effects with measured exposure data. Moreover, categorization of subjects as normal or outlier allowed the evaluation of the association between occupational exposure to PAH and DNA damage highlighting an increased genotoxic risk.

## Background

Graphite electrodes are used in the metal industry, mainly in electric arc furnace steel production, to refine steel in ladle furnaces, and in other smelting processes [1]. The production process of graphite electrodes involves the use of coal tar, coal tar pitch and petroleum coke with workers possibly exposed to polycyclic aromatic hydrocarbons (PAH) by inhalation (of PAH both volatile and bound to respirable particulate matter) and dermal contact [2]. Occupational exposure of workers to PAH-containing coal tar pitch volatiles, pitch and coke occurs during the manufacturing process particularly during the heating of raw materials [3-6]. Occupational exposure to PAH was associated with increased risk of developing lung, skin, bladder and prostate cancer among graphite electrode manufacturing workers [2,7-10].

Several hundred PAH have been characterized for their chemistry and many individual PAH (*e.g. *benzo [a]pyrene, benzo [a]anthracene, dibenzo [a, h]anthracene) are regarded as probably or possibly carcinogenic to humans by the International Agency for Research on Cancer (group 2A or 2B) [11]. The carcinogenic activity of PAH is related to the DNA damaging activity of some of their metabolites which can covalently bind to nucleophilic residues of DNA bases. The PAH are activated to the corresponding electrophilic diolepoxides (ultimate carcinogens) via cytochrome P450 (CYP) enzyme system [12]. The inactivation of diolepoxide metabolites occurs mainly through conjugation with reduced glutathione (GSH) by glutathione *S*-transferase (GST) enzymes [13].

Many genes encoding carcinogens metabolising enzymes have been found to be polymorphic in humans, with relevance to the individual response to carcinogens, probably acting as modifiers of exposure biomarkers (susceptibility markers) [14-16].

A critical polymorphic gene which contributes to the bioactivation of many PAH is the *CYP1A1 *encoding the cytochrome P4501A1 enzyme (CYP1A1), an inducible enzyme with aryl hydrocarbon hydroxylase activity. Among several polymorphisms identified in the *CYP1A1 *gene, two closely linked mutations have been extensively studied in relation to cancer risk. The *CYP1A1 *Ile/Val (*m2*) mutation in the heme-binding region doubles the microsomal enzyme activity and it is in linkage disequilibrium in Caucasians with the *CYP1A1 Msp*I (*m1*) mutation, that has also been associated experimentally with increased catalytic activity [17]. Positive associations between the presence of these variant alleles and increased PAH-DNA adducts have been reported [18-20].

A further critical enzyme in PAH metabolism is microsomal epoxide hydrolase (mEH) which catalyses the hydrolysis of epoxides into dihydrodiols, and as such plays an important role in the detoxification of toxic, highly reactive, intermediates formed by cytochrome P450-mediated reactions [21]. Low mEH activity has been associated with adverse drug responses or diseases states [22]. Two polymorphic sites within the mEH gene (*EPHX*) have been identified. A substitution at codon 113 (Tyr → His) in exon 3 is associated with decreased mEH activity, whereas a substitution at codon 139 (His → Arg) in exon 4 is associated with increased mEH activity. These mutations affect mEH enzyme activity by altering protein stability without affecting the specific activity [23].

The *GSTM1 *gene encodes for the cytosolic enzyme glutathione *S*-transferase μ1 (GSTM1) that detoxify activated forms of chemical carcinogens such as polyaromatic hydrocarbon epoxides. This gene is deleted in about 50% of Caucasians, with a reported variation of from 38 to 65% [24]. The inherited absence of the *GSTM1 *gene (the *GSTM1 *null genotype) is therefore theoretically associated at a higher risk to the toxic effects of chemicals and its influence on various biomarkers of exposure has been widely studied [18,25-27].

In the present paper we reported the results of a cross-sectional study aimed to evaluate whether genetic polymorphisms (biomarkers of susceptibility) for *CYP1A1*, *EPHX *and *GSTM1 *genes that affect PAH activation and detoxification might influence the extent of primary DNA damage (biomarker of biologically effective dose) in PAH exposed workers (*n *= 109) and in unexposed controls (*n *= 82). PAH-exposure of the study populations was assessed by determining the concentration of 1-hydroxypyrene (1OHP) in urine samples (biomarker of exposure dose). The urinary concentration of 1OHP, a metabolite of the non carcinogenic PAH pyrene, is a well validated marker for PAH-exposure [28] giving an accurate assessment of total PAH exposure from all exposure routes [29]. The extent of primary DNA damage was evaluated with the comet assay in peripheral blood leukocytes (PBL) [30]. The study subjects were genotyped for polymorphisms in the *CYP1A1 *gene (mutations associated with increased catalytic activity) encoding the cytochrome P4501A1 enzyme, in the *EPHX *gene (mutations influencing the mEH enzyme activity) encoding for the mEH enzyme, and the *GSTM1 *gene (inherited absence) encoding for the cytosolic glutathione *S*-transferase μ1 (GSTM1).

## Methods

### Study Population and Samples Collection

A total of 191 healthy men living in the same area of Central Italy were enrolled in the study. The exposed group was composed of 109 workers at a graphite electrode producing plant, occupationally exposed to PAH. The electrode plant produces large electrodes (length 2.5÷3 m, diameter up to 1 m) and small bricks (diameter less than 0.1 m) of electrode paste for steel industry [3]. As a control group, 82 reference subjects (not occupationally exposed to PAH) were recruited from the technical and maintenance staff of the University of Perugia.

Workers and reference subjects were interviewed to obtain personal data and information on current job, smoking habit, alcohol consumption, diet, and current and past health status (including information on medicine intake, X-ray examinations, and viral infections). Following informed consent was obtained from all individuals enrolled in the study, workers and reference subjects provided a urine sample for 1OHP determination and a peripheral venous blood sample (using heparinized vacutainer tubes) for the comet assay and genotyping. Biological samples were collected from workers at the end of the shift after at least 4 consecutive days at work. The samples were coded and immediately transferred to the laboratory in refrigerated boxes.

The research protocol was approved by the Ethics Committee of the University of Perugia.

### *CYP1A1*, *EPHX*, and *GSTM1 *Genotyping

Total DNA was extracted from peripheral blood cells using standard techniques. All genotypes were determined after gene amplification using polymerase chain reactions (PCR) [31]. The T → C mutation (*m1*) in the 3'-flanking region of *CYP1A1 *gene was detected by PCR followed by restriction fragment length polymorphism (RFLP) analysis using the restriction enzyme *Msp*I [32]. The genotype w_1_/w_1 _(lacking the *Msp*I site) forms an uncleaved 340 bp band, while the genotype m_1_/m_1 _(homozygous for the allele carrying the mutation with the *Msp*I site) generates two bands of 200 and 140 bp. The heterozygous genotype m_1_/w_1 _corresponds to three bands of 340, 200, and 140 bp.

The *CYP1A1* Ile/Val replacement (*m2*) was detected by *Bsr*DI-RFLP analysis [33]. The Ile/Val polymorphism arises from a A → G base change resulting in the replacement of isoleucine by valine at residue 462 in the heme binding region of the enzyme. The Val allele variant shows an almost two-fold higher catalytic enzyme activity than Ile form.

Amplicons of exons 3 and 4 of *EPHX *gene (162, and 381 bp, respectively) were obtained by PCR, RFLP digestions were then performed to determine the exon 3 (Tyr113His) and exon 4 (His139Arg) genotypes, using the restriction enzymes *EcoR*V and *Rsa*I, respectively [31]. On the basis of the polymorphisms at codon 113 (exon 3) and 139 (exon 4) of *EPHX *gene, the subjects were classified according to expected mEH enzyme activity (low mEH, intermediate mEH, or high mEH activity) [31].

*GSTM1 *genotyping for gene deletions was carried out by detecting the presence or the absence of the intact gene [34]. The absence of *GSTM1 *specific amplification products revealed the corresponding null genotype (homozygous deletion of the *GSTM1 *gene, resulting in deficiency of GSTM1 activity). The *GSTM1 *positive genotype, detected by the presence of *GSTM1 *specific band of 215 bp, contained wild-type homozygotes and heterozygotes for the deletion (not differentiated in the analysis), both expressing GSTM1 enzyme. Co-amplification of β-globin gene was used as an internal control (presence of amplifiable DNA in the sample).

### Analysis of 1-Hydroxypyrene in Urine

Urinary concentrations of 1OHP were determined by HPLC in enzymatically hydrolyzed urine samples [28]. Urine samples, adjusted to pH 5.0, were treated overnight at 37°C with β-glucuronidase and aryl sulfatase and then purified with solid phase extraction with Sep-Pack C18 cartridges primed with methanol. The cartridges were then washed with high purity water and 1OHP was eluted with methanol. The eluate was gently evaporated to dryness under nitrogen and reconstituted in methanol. Of the reconstituted eluate, 15 μl were injected into an HPLC and 1OHP, eluting at a retention time of 8 min, was detected with excitation and emission wavelengths of 347 and 388 nm, respectively.

### Analysis of Primary DNA Damage (Comet Assay) in Leukocytes

PBL were obtained from whole blood by lysis of erythrocytes [35]. Viability of cells after isolation was determined by the fluorochrome-mediated (simultaneous staining with fluorescein diacetate and propidium iodide) viability test [36]. Isolated PBL were processed in the comet assay following the standard alkaline protocol [37], with minor modification [38,39].

The cells (2 × 10^5^) were mixed with 0.7% low melting temperature agarose (total volume 75 μl/slide) and sandwiched between a layer of 0.5% normal melting temperature agarose (75 μl) and a top layer of 0.7% low melting temperature agarose (65 μl) onto conventional microscope slides. Lysis of cellular and nuclear membranes of the embedded cells was performed by immersing the slides for 60 min, at 4°C in the dark, in ice-cold freshly prepared lysis solution (10 mM Tris-HCl, 1% sodium *N*-lauroylsarcosinate, 2.5 M NaCI, 100 mM Na_2_EDTA, 1% triton X-100, and 10% DMSO; pH 10). The slides were removed from the lysis solution and then placed on a horizontal electrophoresis box. The unit was filled with freshly made alkaline buffer (300 mM NaOH, 1 mM Na_2_EDTA; pH > 13) to a level of 0.25 cm over the slides. To allow DNA unwinding and expression of alkali labile damage, the embedded cells were exposed to alkali for 20 min, then the electrophoresis was performed in the same buffer for 20 min by applying an electric field of 25 V (1 V/cm) and adjusting the current to 300 mA.

To control the assay conditions, particularly slides preparation procedure and electrophoresis efficiency, negative and positive internal controls (Jürkat cells, human lymphoblastoid T-cells) were processed in parallel with whole blood samples. Jürkat cells were untreated (negative control) or incubated for 1 h with 1 μg/ml 4-nitroquinoline-*N*-oxide (positive control). Electrophoresis runs were considered valid only if the internal controls yielded the expected results.

After electrophoresis, the slides were first washed gently with 0.4 M Tris-HCl buffer (pH 7.5) to neutralize the alkali, and the DNA was then stained by adding 100 μl of ethidium bromide (2 μg/ml). The slides were kept in a humidified sealed box to prevent drying of the gel and analyzed within 48–72 hours.

Comets in each gel were analyzed (blind) at 500× magnification using an epi-fluorescent microscope (excitation filter, 515–560 nm; barrier filter, 590 nm) equipped with a high sensitivity black and white CCD camera. For each subject, the average tail length, tail intensity and tail moment values were determined scoring 150 comets (50 comets/slide, from at least three replicate slides). Imaging was performed using a specialized analysis system ("Comet Assay III", Perceptive Instruments).

### Statistical Analysis of Data

The analyses were carried out using the SPSS 10.0 statistical software package (SPSS Inc., IL, USA). For each subject, the averaged migration extents (i.e. tail length, tail intensity, and tail moment) of the 150 cells analyzed were used as a data point in data analysis. Cells were also classified as either "undamaged" or "damaged" by considering threshold levels indicating the cells with abnormal size tail (AST) (*i.e. *the 95th percentile of the distribution of the tail parameters among controls) [40]. Cells with tail parameters values below the cut-off (18.87 μm, 16.73%, and 1.90 for tail length, tail intensity, and tail moment, respectively) were classified as "undamaged", and those with higher values as "damaged". Based on these binary outcomes, each subject was classified as "normal" or "outlier" by considering the upper bound of the expected number of AST which was calculated for each subject from the binomial distribution [41]. The latter outcome variable (i.e. each subject defined as normal or outlier) is again binary, becoming the subject the statistical unit.

Group differences in concentration of 1OHP and extent of primary DNA damage (individual averaged tail parameters) were tested with the non-parametric Mann-Whitney *U*-test (two-tailed). The Hardy-Weinberg equilibrium test for genotype distribution was performed using a χ^2 ^test with 1 degree of freedom. The *GSTM1 *genotype was coded as positive (wild-type homozygotes and heterozygotes for the deletion) or null (homozygous deletion), making direct calculation of Hardy-Weinberg equilibrium impossible. The Pearson-χ^2 ^test was used to determine significant differences in the distribution of outliers subjects and of allele frequencies of the considered genotypes among the groups. Outlier subjects prevalence odds ratio (OR) with 95% confidence intervals (95% CI) were calculated by means of cross tabulation. The level of statistical significance was set at *p *< 0.05.

## Results

### Characteristics of the Study population

Demographic characteristics of the study population, also grouped according to exposure status and smoking habits, are reported in Table 1. With respect to age and smoking habits, exposed and control groups were comparable. The exposed workers and controls were stratified by genotypes, *CYP1A1 Msp*I (*m1*), *CYP1A1 *Ile/Val (*m2*), *EPHX *and *GSTM1 *(Table 2). A similar frequency distribution (Pearson-χ^2 ^test) was observed in the groups for the considered genotypes. With regard to the *CYP1A1 *gene, 38 (20.2%) subjects resulted heterozygotes for the *Msp*I mutant allele, 27 (24.8%) exposed workers and 11 (13.8%) controls. The 5 (2.6%) *Msp*I homozygotes individuals, 3 (2.8%) exposed workers and 2 (2.5%) controls, were combined with heterozygous subjects for subsequent statistical analyses. In the group of 189 men examined, the mutant Val allele for *CYP1A1 *gene occurred in 17 (9.0%) individuals, 11 (10.1%) exposed workers and 6 (7.5%) controls. Only 1 (0.5%) person (control subject) resulted to be a carrier of the homozygous Val/Val variant of *CYP1A1 *and was combined with heterozygous individuals for statistical analysis. About 50% of the studied subjects (94 individuals) showed to have a low mEH deduced activity (*i.e. *mEH activity deduction based on the results of genotyping of *EPHX *polymorphisms in exons 3 and 4), 59 (54.1%) exposed workers and 35 (43.8%) controls. The residual 95 subjects (50.3%), 50 (45.9%) exposed workers and 45 (56.2%) controls, showed to have a medium/high mEH deduced activity. The above reported genotypes (i.e. *CYP1A1 Msp*I (*m1*), *CYP1A1 *Ile/Val (*m2*), *EPHX*), among exposed and control subjects were in Hardy-Weinberg equilibrium (data not shown). In the whole study population, the prevalence of *GSTM1 *null subjects was 82 (43.4%), of which 46 (42.2%) exposed and 36 (45.0%) controls. Only 21 (11.1%) subjects were carriers of the *GSTM1 *null + *CYP1A1 *w_1_/m_1 _+ m_1_/m_1 _combined genotype, 13 (11.9%) exposed and 8 (10.0%) controls; 10 (5.3%) subjects presented the *GSTM1 *null + *CYP1A1 *w_2_/m_2 _+ m_2_/m_2 _combined genotype, 5 (4.6%) exposed and 5 (6.3%) controls; 9 (4.8%) individuals were carriers of the susceptible *GSTM1 *null + *CYP1A1 *w_1_/m_1 _+ m_1_/m_1 _+ *CYP1A1 *w_2_/m_2 _+ m_2_/m_2 _genotype, 5 (4.6%) exposed and 4 (5.0%) controls.

**Table 1:** Demographic characteristics of the study population grouped according to exposure status.

	Exposed workers	Controls
Subjects	109	82
Demographic characteristics^a^		
Age^b^	43.32 ± 0.56	42.99 ± 0.73
≤ 40 years	34	30
> 40 years	75	52
Occupational features		
Duration of employment^b^	14.34 ± 8.05	---
≤ 10 years	36	---
> 10 years	73	---
Smoking habits		
Non smokers	54	42
Smokers	55	40
Cigarettes/day^c^	18.85 ± 9.14	14.92 ± 8.11

^a ^All the subjects were males.

^b ^Age and duration of employment are expressed in years and reported as the group mean ± standard deviation.

^c ^The number of cigarettes smoked per day is reported as the mean ± standard deviation.

**Table 2:** Distribution of *CYP1A1*, *EPHX*, and *GSTM1 *genotypes in exposed and control subjects. Data are reported as the number of subjects (percentage between brackets).

Genotype	Exposed workers	Controls^a^
*CYP1A1 *(*Msp*I)^b ^*w1*/*w1*	79 (72.5)	67 (83.8)
*w1*/*m1 *+ *m1*/*m1*	30 (27.5)	13 (16.3)
*CYP1A1 *(Ile/Val)^c ^*w2*/*w2*	98 (89.9)	73 (91.3)
*w2*/*m2 *+ *m2*/*m2*	11 (10.1)	7 (8.8)
*EPHX*^d ^Low	59 (54.1)	35 (43.8)
Medium + High	50 (45.9)	45 (56.3)
*GSTM1 *Active	63 (57.8)	44 (55.0)
Null	46 (42.2)	36 (45.0)

^a ^Two control subjects were not genotyped.

^b ^*w1 *= common allele, *m1 *= variant allele.

^c ^*w2 *= common allele; *m2 *= variant allele.

^d ^mEH-deduced activity. The mEH activity deduction was based on the results of genotyping of polymorphisms in exons 3 and 4: subjects carrier of combination (exons 3 and 4) His113His + His139His, His113His + His139Arg, or Tyr113His + His139His, were considered as having "low" activity of mEH; subjects carrier of combination (exons 3 and 4) Tyr113Tyr + His139His, Tyr113His + His139Arg, or His113His + Arg139Arg, were considered as having "medium" activity of mEH; subjects carrier of combination (exons 3 and 4) Tyr113Tyr + His139Arg, Tyr113Tyr + Arg139Arg, or Tyr113His + Arg139Arg, were considered as having "high" activity of mEH [31].

### Influence of Exposure on the Biological End-Points

Statistically significant correlations were found between the three different parameters measuring the extent of DNA damage in leukocytes (i.e. tail length, tail intensity and tail moment), thus data presentation will be limited to tail intensity, also taking into account that relative tail intensity (percentage of DNA migrated in the comet tail) is considered to be the most useful parameter, as it bears a linear relationship to strand-breaks frequency and is relatively unaffected by threshold settings in the computerized analysis system [42].

Group mean values (± SEM) of urinary 1OHP, individual averaged percentage of DNA migrated in the comet tail (*i.e. *tail intensity) and number of AST are listed in Table 3; the proportions of outlier subjects in exposed and control groups (*i.e. *tail intensity) and the OR values are showed in Table 4.

**Table 3:** Urinary concentration of 1-hydroxypyrene (1OHP) and extent of primary DNA damage in peripheral blood leukocytes in exposed workers and control subjects. Data reported as the group mean values (± SEM) of individual: urinary concentration of 1OHP (expressed as μg 1OHP/g creatinine), averaged tail intensity values (% of DNA migrated in the comet tail evaluated in 150 cells) and number of AST.

	1OHP	DNA damage	
		
		Averaged counts	AST
*Exposed*			
Total	2.64 ± 0.29*	5,28 ± 0,21*	11,75 ± 0,81*
≤ 40 years	2,96 ± 0,43*	4,63 ± 0,36	9,59 ± 1,35
> 40 years	2,49 ± 0,37*	5,58 ± 0,26*	12,73 ± 0,99*
Employment ≤ 10 years	2,23 ± 0,34	4,90 ± 0,29	10,14 ± 1,16
Employment > 10 years	2,84 ± 0,40	5,47 ± 0,28	12,55 ± 1,05
Non smokers	2.85 ± 0.47*	5.47 ± 0.30*	12,81 ± 1,14*
Smokers	2.43 ± 0.34*	5.11 ± 0.31	10,71 ± 1,14*
*Controls*			
Total	0.15 ± 0.02	4,33 ± 0,22	7,50 ± 0,98
≤ 40 years	0.17 ± 0.03	4.34 ± 0.35	6,83 ± 1,51
> 40 years	0,14 ± 0,02	4,33 ± 0,28	7,88 ± 1,29
Non smokers	0.10 ± 0.02	4.23 ± 0.31	8,36 ± 1,60
Smokers	0.20 ± 0.02^§^	4.44 ± 0.32	6,60 ± 1,12

* *p *< 0.05, exposed *vs*. controls; ^§ ^*p *< 0.05, non-smokers *vs*. smokers. Non-parametric Mann-Whitney *U*-test (two-tailed).

**Table 4:** Proportions of outlier subjects in exposed and control groups. Data are referred to tail intensity (% of DNA migrated in the comet tail).

	Exposed	Controls	OR (95% CI)
Total	42*	16	2.59 (1.32–5.05)
≤ 40 years	11	5	2.39 (0.72–7.93)
> 40 years	31*	11	2.63 (1.17–5.90)
Employment ≤ 10 years	15		
Employment > 10 years	27		
Non smokers	24*	9	2.93 (1.18–7.30)
Smokers	18	7	2.29 (0.85–6.18)

* *p *< 0.05, exposed *vs*. controls. Pearson-χ^2 ^test.

Urinary 1OHP was significantly higher in electrode workers than in reference subjects, with 1OHP concentrations about 18-fold higher in exposed workers than in controls (*p *< 0.001). Smoking resulted in a statistically significant increase of the 1OHP levels in controls (*p *< 0.001), but not in the exposed group. Moreover, urinary 1OHP was significantly lower (*p *= 0.046) in electrode workers older than 40 respect to the youngest subjects. Urinary 1OHP concentrations found in the present study correlate (*r *= 0.732) with the exposure to total PAH (data not shown).

Factory workers showed a statistical significant increase in averaged DNA damage over that for controls (*p *= 0.002). Smoking habit did not increase the extent of DNA damage, both in the exposed and in the reference groups. Primary DNA damage was significantly higher (*p *= 0.036) in electrode workers older than 40 respect to the youngest subjects.

The group mean value of damaged cells (AST) is significantly higher in the exposed than in control subjects (*p *< 0.001). Moreover, the number of 'outlier' subjects (subjects showing a high number of AST) is significantly higher in the exposed (38.5%) than in the control group (19.5%), with an OR = 2.59 (CI95% 1.32–5.05). In the group of exposed workers, the higher frequency of AST and of outliers is mainly accounted by the subjects with an age over 40 years, with an OR = 2.63 (CI95% 1.17–5.90).

### Influence of Genotypes on the Biological End-Points

The influences of genetic polymorphisms on the concentration of urinary 1OHP and the extent of primary DNA damage are shown in Table 5. A statistically significant influence of genetic polymorphisms was observed only between exposed subjects with *EPHX *low or medium + high deduced activity for 1OHP concentration in urine samples.

**Table 5:** Urinary concentration of 1-hydroxypyrene (1OHP) and extent of primary DNA damage in peripheral blood leukocytes in exposed workers and control subjects with respect to metabolic genotypes (*CYP1A1*, *EPHX*, and *GSTM1*). Data reported as the group mean values (± SEM) of individual: urinary concentration of 1OHP (expressed as μg 1OHP/g creatinine), averaged tail intensity values (% of DNA migrated in the comet tail evaluated in 150 cells) and number of AST.

	1OHP	DNA damage	
		
		Averaged counts	AST
*Exposed*			
*CYP1A1 *(*Msp*I)^b ^w_1_/w_1_	2.37 ± 0.29*	5.08 ± 2.45*	11.23 ± 0.92*
w_1_/m_1 _+ m_1_/m_1_	3.33 ± 0.71*	5.81 ± 0.43	13.13 ± 1.65*
*CYP1A1 *(Ile/Val)^c ^w_2_/w_2_	2.71 ± 0.31*	5.22 ± 0.22*	11.51 ± 0.84*
w_2_/m_2 _+ m_2_/m_2_	1.96 ± 0.61*	5.85 ± 0.75	13.91 ± 2.95
*EPHX*^d ^Low	2.17 ± 0.37*,^§^	5.22 ± 0.30	12.63 ± 1.12*
Medium + High	3.19 ± 0.45*	5.00 ± 0.30*	10.72 ± 1.16*
*GSTM1 *Active	2.53 ± 0.38*	5.46 ± 0.29*	12.14 ± 1,09*
Null	2.79 ± 0.45*	5.05 ± 0.32	11.22 ± 1,21*
*Controls*			
*CYP1A1 *(*Msp*I)^b ^w_1_/w_1_	0.14 ± 0.02	4.28 ± 0.25	7.24 ± 1.10
w_1_/m_1 _+ m_1_/m_1_	0.21 ± 0.05	4.87 ± 0.53	6.54 ± 1.30
*CYP1A1 *(Ile/Val)^c ^w_2_/w_2_	0.14 ± 0.02	4.38 ± 0.24	6.77 ± 0.91
w_2_/m_2 _+ m_2_/m_2_	0.20 ± 0.07	4.25 ± 0.30	10.86 ± 5.08
*EPHX*^d ^Low	0.13 ± 0.02	4.58 ± 0.33	7.34 ± 1.13
Medium + High	0.16 ± 0.02	4.21 ± 0.30	6.96 ± 1.43
*GSTM1 *Active	0.15 ± 0.02	4.49 ± 0.32	8.32 ± 1,48
Null	0.15 ± 0.03	4.23 ± 0.31	5.67 ± 1,02

^a, b, c, d ^See notes in Table 2.

* *p *< 0.05, exposed *vs*. controls; ^§ ^*p *< 0.05, *EPHX *Low *vs*. *EPHX *Medium + High. Non-parametric Mann-Whitney *U*-test.

## Discussion

This study was designed to evaluate the occupational exposure to PAH, as assessed by biological monitoring techniques, in workers employed at a graphite electrode manufacturing plant and in reference subjects with presumed lower exposure to PAH. Current exposure to PAH was assessed by determining the urinary concentration of 1OHP (biomarker of internal dose), whereas biological effect monitoring focused on the evaluation of primary DNA damage extent as evaluated by the comet assay in PBL (biomarker of biologically effective dose). Moreover, in this study we analysed genetic polymorphisms in the *CYP1A1 *(*Msp*I and Ile/Val sites), *EPHX *(exons 3 and 4), and *GSTM1 *genes to evaluate the impact of these metabolic genotypes (biomarker of individual susceptibility) on the levels of urinary 1OHP and the extent of primary DNA damage.

Urinary 1OHP levels were significantly higher in exposed workers than in matched controls. This finding is consistent with the results of other studies aimed to determine the effects of occupational exposure to PAH on urinary 1OHP concentrations [3,43-45]. The statistically significant increase in urinary 1OHP levels observed in control smokers as compared to control non smokers was not confirmed in the group of exposed workers. This aspect could be explained in terms of saturating dose, probably due to the large work-related effects, at which no further effect can be seen at higher doses.

In the comet assay, DNA strand breakage is quantified from geometric (*e.g. *migration distance) and fluorescence (*e.g. *per cent of fluorescence migrated in the tail) measurements both by eye (*i.e. *comet score) or computerized image analysis. In the presence of DNA damage, the distribution of tail parameters results strongly skewed to the right and the two sides of the distribution have different spreads. In this situation the mean is strongly influenced by extreme observations and, as the resulting large standard deviation, appears to be rather uninformative. In this situation, skewed not symmetrical distributions could be defined more accurately by the median value (*i.e. *50th percentile) or the values corresponding to the 75th/95th percentile [46]. This statistical approach in the comet assay was adopted in several researches [47-49].

In epidemiological studies, enrolled subjects should be characterized for a given aspect in a simple manner, preferably with a single number. In this molecular epidemiology approach we have chosen to describe the extent of DNA damage only in terms of tail intensity, a parameter describing the percentage of DNA migrated in the tail which is relatively unaffected by threshold settings in the computerized imaging [42].

As regard the extent of primary DNA damage, the comet assay has been employed only in few studies aimed to evaluate the PAH genotoxic effects in exposed workers [45,50-55], with both positive and negative findings. DNA single-strand breakage did not differ between 99 potroom workers at an aluminum reduction plant and 55 unexposed referents [50]. Occupational exposure to PAH did not result in increased DNA strand breaks coke oven workers as compared to unexposed subjects [55]. The analysis of DNA damage did not show significant differences between 42 primary aluminium industry workers and 16 local residents with no occupational exposure to PAH [51]. No effect of occupational exposure was observed in 50 coke oven workers as compared to 50 control workers not exposed to PAH in the extent of DNA damage [45]. Exposure to PAH caused a significantly higher single strand DNA breakage in lymphocytes and granulocytes of 24 workers from automobile emission inspection companies and 28 workers from a waste incinerating company as compared to 43 matched unexposed subjects [54]. The extent of primary DNA damage evaluated with the comet assay was found to be 3.13 times higher for graphite-electrode-producing plant workers (n = 29) when compared with controls (n = 32) [52]. Thus, the findings of the present work are in line with the results of the unique study considering graphite-electrode-producing plant workers [52] and support the evidence that occupational exposure to PAH during graphite electrode manufacturing can result in primary DNA damage (strand breakage as evaluated with the comet assay).

Smoking habit did not increase any of the DNA damage parameters, either in the exposed than in the reference group. The effect of smoking as a potential confounder in occupational studies has been recently evaluated in a meta-analysis study of the available, conflicting results obtained with the comet assay [56]. The authors concluded that an effect of smoking could not be formally demonstrated when the evaluation of DNA damage was based on image analysis.

Homozygous variant carriers of the *CYP1A1 *polymorphisms (*Msp*I and Ile/Val) are extremely rare in Caucasians [57]. The frequencies (all subjects) that we found (2.65 and 0.53%, for *Msp*I and Ile/Val, respectively) agree with these observations. The *EPHX *allele frequencies found in this study are similar to those reported in non-Hispanic whites [58]. The frequency of the *GSTM1 *null genotype (43.4%) among the studied population (all subjects) agrees with the frequencies reported in the literature for the Caucasian population indicating that 40÷50% of the considered subjects lack of GSTM1 activity [59,60].

Our results indicate that polymorphisms in *EPHX *exons 3 and 4 is associated to higher urinary concentrations of 1OHP, whereas the presence of variants in the *CYP1A1 *gene (i.e. *Msp*I and Ile/Val mutations) as well as the presence of the *GSTM1 *null genotype showed to have no effect on urinary 1OHP excretion. The increased urinary excretion of 1OHP in subjects having a high EPHX activity agrees with previously published results [61]. Whereas, the absence of a relationship between urinary levels of 1OHP and the presence of the *GSTM1 *null genotype is not unexpected, as 1OHP is mainly excreted as glucuronide conjugate [62].

None of the genotypes analyzed (*CYP1A1*, *EPHX*, and *GSTM1*) had any significant influence on primary DNA damage as evaluated by the comet assay. However, it was reported that DNA damage by benzo(a)pyrene (*i.e. *benzo [a]pyrene diolepoxide-DNA adducts) in PAH-exposed coke oven workers is influenced by smoking habits and *GSTM1 *polymorphisms [63,64]. Thus, it could be of interest in the future, in workers exposed to high concentration of PAH, to compare damage caused by benzo(a)pyrene (such as BPDE-DNA adducts) and DNA strand breakage (as evaluated with the comet assay) also in relation to genetic polymorphisms.

No significant correlations were observed between urinary levels of 1OHP and the extent of DNA strand breakage (Spearman's correlation coefficients: *r *= 0.338, *r *= 0.157, and *r *= 0.205, for tail length, tail intensity, and tail moment, respectively) in this study. The absence of significant correlations could be explained in terms of different persistence for the considered biomarkers. In fact, urinary metabolites (*i.e. *1OHP) mirror the exposure during the last workshift and some days before [52], whereas the alkaline comet assay measures temporary strand breaks that happen before the DNA repair systems or cell turnover occur [65].

## Conclusion

The results of a cohort study among workers in a graphite electrode production plant in Italy showed an excess of mortality for cancer in these workers with a standardised mortality ratio of 1.27 (CI95% 1.07–1.50) [66]. The main strategy of primary cancer prevention is minimize exposures to recognized genotoxic/carcinogenic risk factors. The outcomes of the present study, together with the results previously published by Marczynski et al. [52], show that molecular epidemiology approaches (*i.e. *cross-sectional studies of genotoxicity biomarkers) can play a role in identifying common genetic risk factors, also attempting to associate the effects with measured exposure data. Moreover, categorization of subjects as normal or outlier, as performed in this study, allowed the evaluation of the association between exposure to genotoxins in this occupational branch and DNA damage, highlighting an increased genotoxic risk with a statistically significant OR = 2.59 (CI95% 1.32–5.05).

## Competing interests

The author(s) declare that they have no competing interests.

## Authors' contributions

GM, RP^1 ^have been involved in the initial design and conception of the study.

MDO was involved in and coordinated all aspects of the study, participated in research design, preparation of study materials and protocol, performed the 1OHP analysis and contributed to interpretation of findings.

MVand MM participated in research design, preparation of study materials and protocol, performed the comet assay.

RP^3^, LA participated in preparation of study materials and protocol, performed the genotype analyses.

MM has been involved in analysis and interpretation of data and conceived and wrote the first draft of the manuscript. MV, MDO, RP^3 ^provided extensive input, feedback and editing on all sections of the paper.

All authors reviewed and approved the final version of this manuscript.

## Pre-publication history

The pre-publication history for this paper can be accessed here:


